# Supplementary material for: Generative artificial intelligence model for simulating structural brain changes in schizophrenia
Source: Front Psychiatry. 2024 Oct 4;15:1437075. doi: 10.3389/fpsyt.2024.1437075 (PMC11486638; doi:10.3389/fpsyt.2024.1437075)
Supplement: Supplementary file 1 [file DataSheet1.pdf]

	COBRE (n = 142)		ABIDE_NYU (n = 184)	
	SZ	HS	ASD	HS (TD)
Characteristic	n=71	n=71	n=79	n=105
Age, mean (SD), year	39.9 (8.2)	36.5 (8.5)	14.5 (7.0)	15.8 (6.3)
Sex, Male/Female	58/13	50/21	68/11	79/26
FIQ, mean (SD)	97.8 (16.8)	106.8 (11.2)	107.9 (16.6)	113.2 (13.1)
VIQ, mean (SD)	103.2 (16.3)	114.7 (11.6)	105.8 (16.1)	113.1 (12.6)
PIQ, mean (SD)	99.8 (16.9)	112.1 (11.6)	108.8 (17.4)	110.1 (13.7)
CPZE, mean (SD)	407.6 (1072.6)			
PANSS, mean (SD)	58.8 (13.8)			
ADOS, mean (SD)			11.30 (4.1)	

Supplementary Table 1: Demographic and clinical information.

	t-value	MNI cordinates			Number of voxels	*p* values uncorrected
		x	y	z		
**Pre > Post**						
Anterior cingulate cortex	5.95	15	47	13	717	< 0.001
Thalamus	5.92	3	-7	-8	180	< 0.001
Orbitofrontal cortex	4.62	15	44	-20	140	< 0.001
Insula	4.48	45	17	-9	79	< 0.001
Temporal pole	4.46	-42	-1	-20	125	< 0.001
Superior temporal gyrus	4.37	-44	-10	-15	21	< 0.001

Supplementary Table 2: Difference in brain regions pre- and post- transformation.

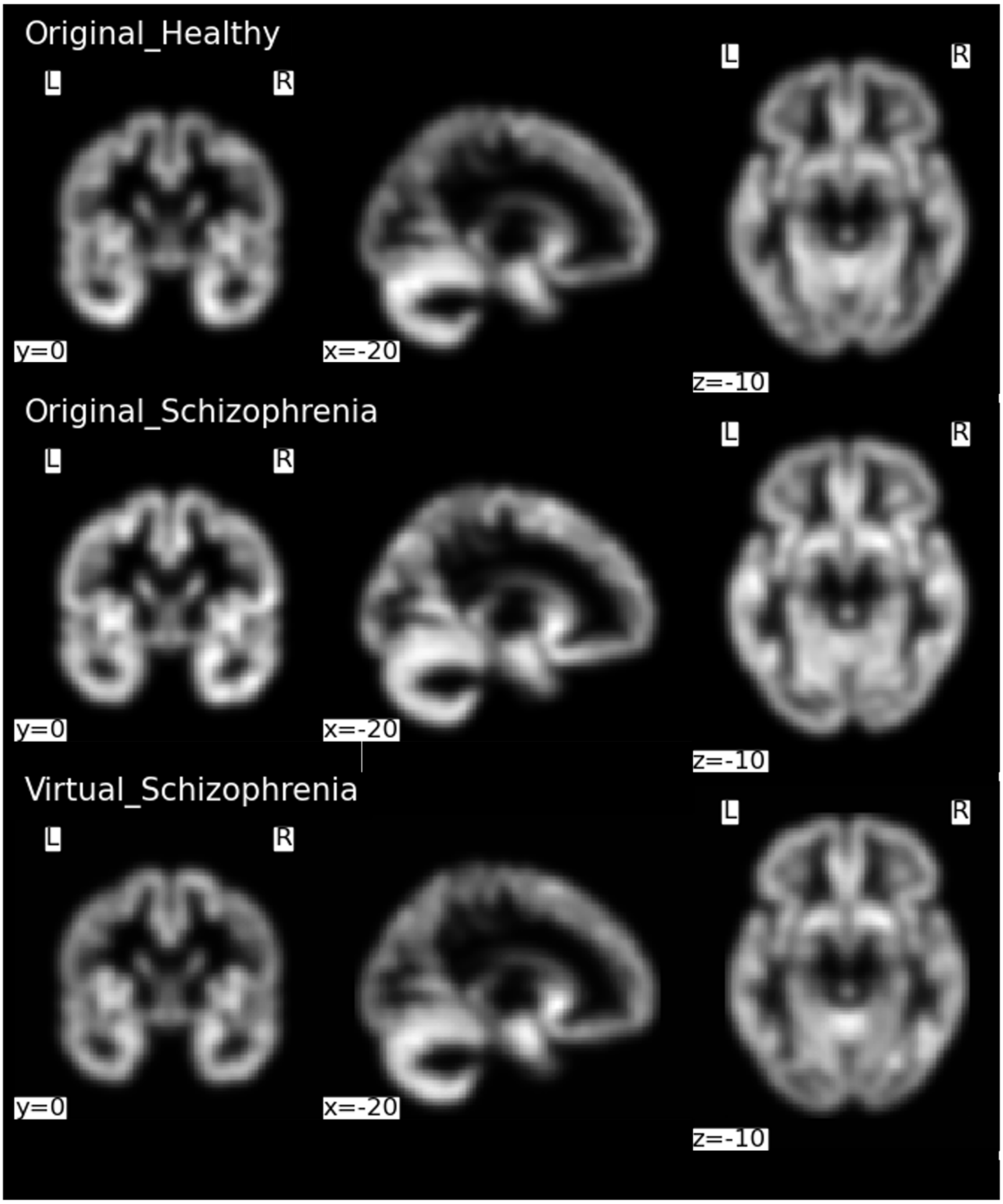

Supplementary Figure 1: Comparison of MRI images of healthy subjects, schizophrenia patients, and generated virtual schizophrenia MRI images.

MRI images of a healthy subject (top row), a real schizophrenia patient (middle row), and a virtual schizophrenia patient (bottom row). Coronal (left), sagittal (middle), and axial (right) views are shown, respectively. A virtual schizophrenia was generated from a healthy subject. It appears qualitatively no different from the real image.

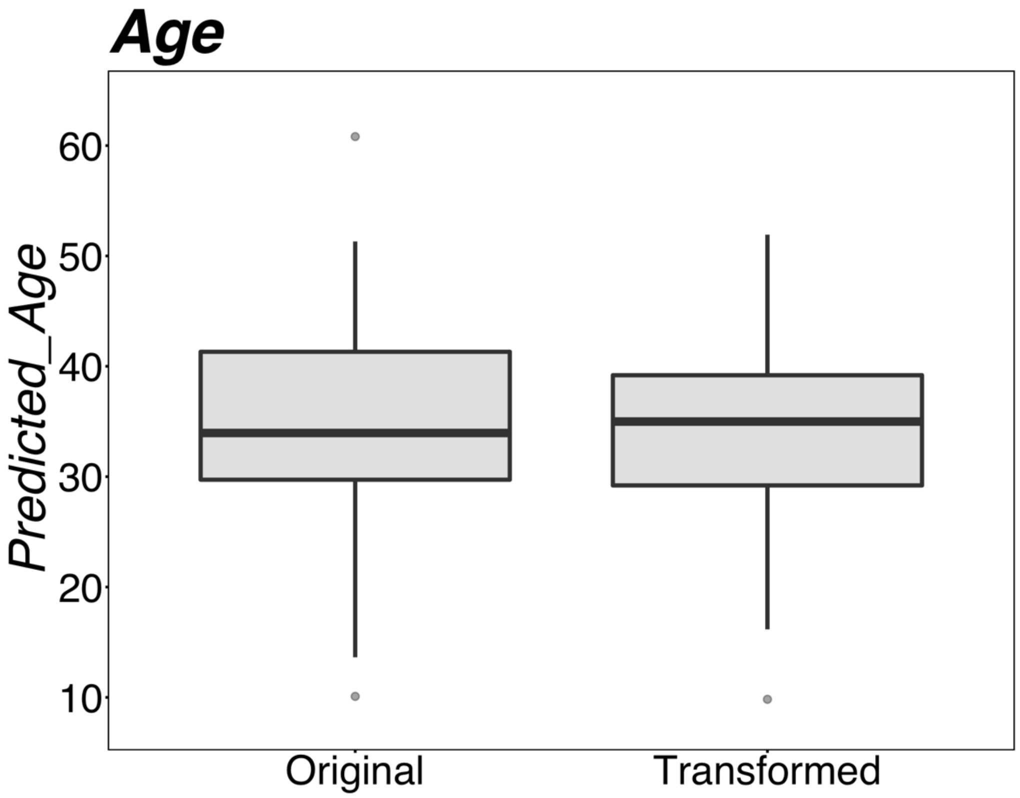

Supplementary Figure 2: Comparison of age predictions pre- and post-transformation

Healthy subject brain MRI was transformed to schizophrenia brain MRI and linear regression was performed for age predictions pre- and post-transformation, respectively. There were no significant differences in predictive values pre- and post-transformation.

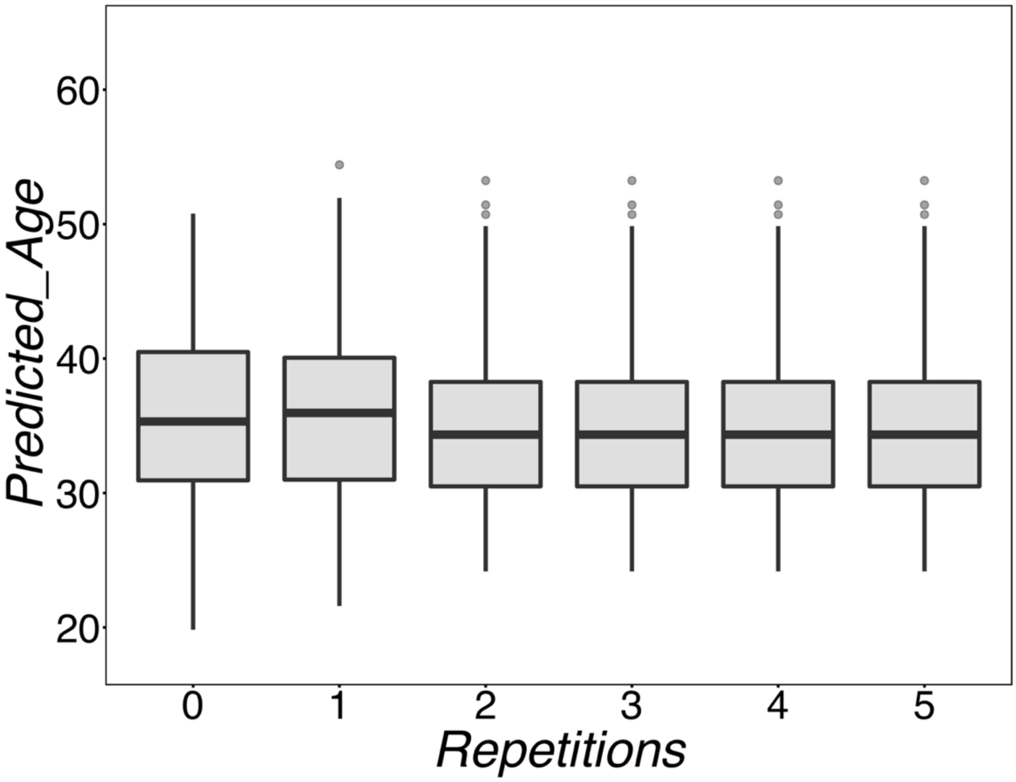

Supplementary Figure 3: Comparison of age prediction by repeatedly transformed brain images

Repeated transformation experiments were performed using the schizophrenia transformer. A linear regression was performed to predict age by each transformed image, and an ANOVA showed no significant difference in predicted values.
